# Artificial light at night decreases plant diversity and performance in experimental grassland communities

**DOI:** 10.1098/rstb.2022.0358

**Published:** 2023-12-18

**Authors:** Solveig Franziska Bucher, Lia Uhde, Alexandra Weigelt, Simone Cesarz, Nico Eisenhauer, Alban Gebler, Christopher Kyba, Christine Römermann, Tom Shatwell, Jes Hines

**Affiliations:** ^1^ German Centre for Integrative Biodiversity Research (iDiv) Halle-Jena-Leipzig, 04103 Leipzig, Germany; ^2^ Department of Plant Biodiversity, Institute of Ecology and Evolution with Herbarium Haussknecht and Botanical Garden, Friedrich Schiller University Jena, 07743 Jena, Germany; ^3^ Systematic Botany and Functional Biodiversity, Institute of Biology, Leipzig University, 04109 Leipzig, Germany; ^4^ Interdisciplinary Geographic Information Sciences, Ruhr-Universität Bochum, 44780 Bochum, Germany; ^5^ Remote Sensing and Geoinformatics, Deutsches GeoForschungsZentrum GFZ, Germany; ^6^ Department of Lake Research, Helmholtz-Centre for Environmental Research – UFZ, 39114 Magdeburg, Germany

**Keywords:** ecotron facilities, global change, light pollution, moonlight, plant functional traits

## Abstract

Artificial light at night (ALAN) affects many areas of the world and is increasing globally. To date, there has been limited and inconsistent evidence regarding the consequences of ALAN for plant communities, as well as for the fitness of their constituent species. ALAN could be beneficial for plants as they need light as energy source, but they also need darkness for regeneration and growth. We created model communities composed of 16 plant species sown, exposed to a gradient of ALAN ranging from ‘moonlight only’ to conditions like situations typically found directly underneath a streetlamp. We measured plant community composition and its production (biomass), as well as functional traits of three plant species from different functional groups (grasses, herbs, legumes) in two separate harvests. We found that biomass was reduced by 33% in the highest ALAN treatment compared to the control, Shannon diversity decreased by 43% and evenness by 34% in the first harvest. Some species failed to establish in the second harvest. Specific leaf area, leaf dry matter content and leaf hairiness responded to ALAN. These responses suggest that plant communities will be sensitive to increasing ALAN, and they flag a need for plant conservation activities that consider impending ALAN scenarios.

This article is part of the theme issue ‘Light pollution in complex ecological systems’.

## 1. Introduction

Artificial light at night (ALAN) affects many areas of the world, especially the northern hemisphere. It is a growing issue, as ALAN is estimated to be globally increasing at rates of 1.6–9.6% per year [1–3]. More than 80% of the human population and 99% of humans in Europe and North America live under light-polluted skies, which influence 88% of Europe's land surface and almost 50% of North America [4]. Ecological effects of light can occur either via direct exposure to lights in urban areas or indirectly via skyglow, i.e. light pollution originating from scattered artificial light from cities, which is detectable in remote places hundreds of kilometres away from urban centres [5]. Intensities of ALAN can be high; the luminance of individual light-emitting elements can be close to daylight conditions, and skyglow can be as strong as light under a full moon [6].

Light exposure presents an important and confounding issue for plants, which rely on light cues for timing of physiological processes and life cycle events (circadian, *ca* monthly and circannual cycles). Natural fluctuations in day length as well as daily and lunar cycles are the most conserved external triggers for plants and animals to perceive time and seasonality and they have been relatively constant throughout evolutionary history—until now [7–10]. Long-term monitoring has revealed that ALAN influences the behaviour of animals, including humans, as their circadian clocks lack external synchronization [8,11–13]. However, most taxa have been poorly studied, and this is especially the case for plants.

Light serves as a fundamental resource for photosynthesis and as a key source of information for plants to time growth and recovery, as well as life-history events such as the onset of flowering or hardening in winter [6,7,9,14–16]. Previous studies have also shown that ALAN can affect production of plant biomass. For example, one of the few long-term experimental studies testing the effects of night light on semi-natural grasslands showed strong species-specific responses in plant biomass and plant cover in the dominant species between years [17]. There might also be an impact on photosynthesis, which could be potentially triggered by exceptional bright ALAN exposure [18]. However, it is necessary for plants to prevent photooxidative damage and repair mechanisms are active during night, which might be hampered by ALAN. Considered together, ALAN may profoundly affect the physiology, composition and functioning of entire plant communities [6,7,14,17,19]. Notably, much of what we know about the effects of ALAN is from case studies on a few plant species and individuals grown in pots rather than studies with a larger numbers of plant species or at the plant community level. Therefore, the responses of individual plants grown in communities as well as of plant communities themselves to increasing ALAN are therefore still not fully understood and rarely experimentally evaluated.

To test the effect of night light exposure on plant community biomass production, diversity and performance, we experimentally manipulated ALAN for a model grassland community sown in the iDiv Ecotron facility [20,21]. Therefore, for three focal species, we assessed individual plant performance by measuring 10 leaf plant functional traits that indicate whether plants benefit from the additional light or are stressed [22–24]. More specifically, we studied morphological traits such as plant height, specific leaf area (SLA), leaf dry matter content (LDMC) and hairiness of the leaves, as well as their thickness, toughness and wettability, along with physiological traits indicating functional processes such as chlorophyll fluorescence (PI_abs_ and F_v_/F_m_) and the chlorophyll content (single-photon avalanche diode, SPAD). Considered together, these 10 plant functional traits provide a comprehensive and mechanistic understanding of plant performance and behaviours under varying ALAN intensities, and whether they are beneficial or detrimental (table 1).

**Table 1.** Plant trait recorded and ecological significance.

plant trait	abbreviation	unit	ecological significance
plant height	—	cm	competitive strength, resource competition for light [23]
specific leaf area	SLA	cm^2^ g^−1^	proxy for growth rate [23,25]
leaf dry matter content	LDMC	mg g^−1^	plant resistance to stress, pathogens and herbivory and slow growth [23,25]
hairiness/ density of trichomes	—		protection of the leaves against excess irradiation [26]
leaf thickness		Mm	drought and heat tolerance [27]
leaf toughness		N mm^−1^	protection from herbivores and physical disturbance, enhances leaf lifespan [28]
wettability	—	(°)	leaf's ability to retain water on its surface [29,30] favours pathogen growth [31] and increases a plant's biochemical stress [32]
chlorophyll fluorescence	F_v_/F_m_		plant stress [15,33,34]
	PI_abs_		performance index, scales with photosynthesis [15,33,34]
chlorophyll content, single-photon avalanche diode	SPAD		SPAD scales with chlorophyll content [35,36]

We thus tested the response of plant community biomass production, diversity, and performance assessed via trait expression to a gradient of simulated ALAN. We asked the following research questions in particular:

1. Is plant community productivity (i.e. biomass production, species composition and diversity) affected by ALAN?
2. Does ALAN influence the expression of traits such as plant height plant traits, reflecting performance of plants within communities?

## 2. Material and methods

### (a) iDiv Ecotron and experimental set-up

The iDiv Ecotron is an indoor mesocosm facility located at the Helmholtz Centre for Environmental Research (UFZ) in Bad Lauchstädt, Germany. Overall, the experiment consisted of 12 EcoUnits, i.e. individual experimental chambers, with controlled abiotic conditions. Each EcoUnit includes both a below-ground compartment with a soil volume of 1.24 × 1.24 × 0.80 m and an above-ground compartment of 1.46 × 1.46 × 1.50 m [21]. The Ecotrons have the advantage of allowing the manipulation of one environmental variable, namely ALAN, while keeping all the other factors constant between EcoUnits. The precipitation regime was set to typical seasonal growth conditions for the region and the sown species (electronic supplementary material, table S1): the mean temperatures of the soil at 20 cm depth between May and December was 17.3°C, with a maximum of 19.4°C in August and a minimum of 15.2°C in May. The experiment was established in February 2020 and the experimental phase of this study started in September 2020.

### (b) Light regime

We established a gradient in ALAN (from no supplemental light to 30.313 lx in the most light-polluted treatment—an illuminance as large as that at pavement level directly underneath a streetlight) between the EcoUnits. The background illumination within the Ecotron facility was 1.4 mlx due to technical devices. The EcoUnits were surrounded by black fabric to avoid light spillage from one unit to another. Additionally, all EcoUnits were exposed to a simulated 28-day moonlight cycle (ranging between 1.4 and 113.7 mlx). All EcoUnits were subjected to a light : dark day length regime that was typical of natural conditions at Bad Lauchstädt (sunrise progressively between 5.07 and 7.32, sunset progressively between 21.23 and 18.20 during the study period; both phases were gradual, allowing 2 h for the transition). Daylight was established using four lamps per EcoUnit, which provide a PAR (photosynthetic active radiation) of about 350 µmol m^−2^ s^−1^ near soil surface (Roschwege GmbH, Greifenstein, Germany). A nine-level ALAN treatment gradient of 0.0087, 0.028, 0.081, 0.103, 0.3, 0.94, 3.033, 9.883, 30.313 lx plus a control of no added light were used following a light intensity gradient on a log scale. Moonlight and additional light treatment were provided by two individually controlled light sources of different LED types (moon: SunLike3030 by Seoul Semiconductor Co. Ltd. Korea; ALAN: type 2835 by HuiYuan Opto-Electronic Co. Ltd. China) combined into one self-made luminaire per EcoUnit. All luminaires were technically identical, and we checked the light regimes via a camera (Canon EOS 6D Mark II + Ex DG Fisheye 8 mm) with high and multiple exposures rate and converted these values to lux using the software Sky Quality Camera 1.9.4. The skyglow started between 18.00 and 21.00 and ended between 6.00 and 8.00 depending on the sun during the study period. The highest and the lowest ALAN treatments were repeated twice, so that overall, we used 12 EcoUnits with 10 different ALAN settings.

### (c) Plant community sowing, growing and biomass harvest

The EcoUnits were filled with 1.23 m^3^ of unsterilized, well-mixed soil from the vicinity of the EcoTron, as we also monitored soil communities in the same experimental set-up [37]. Plant communities comprising 16 plant species were sown into soil on 19 February 2020 (see electronic supplementary material, table S1). Because the soil was not sterilized, some of the local seed bank was also transferred into our experiment. Plant communities were harvested by clipping above-ground plant biomass (2 cm above topsoil) on 11 June, 3 July and 28 August (establishment period) as well as on 27 October and 8 December (measurement period). This harvest regime mimics typical intensive grassland management in central Europe, with short growth phases in between harvest events [38]. For this study, we analysed the last two harvests in detail to address temporal variations and accumulated effects of the ALAN treatment (see electronic supplementary material, table S1; hereafter referred to harvest 1 and 2, respectively). The harvests differed in length: harvest 1 encompassed a time for regrowth of nine weeks, whereas harvest 2 only encompassed six weeks, as this was embedded in a bigger experimental set-up. The biomass of one-eighth (0.19 m^2^) of each EcoUnit (subplot) was separated into species (both sown and not sown, as well as ‘unknown’) and then dried to constant weight at 60°C for 3 days. Plant identification was sometimes not possible when the plants were not fully mature. These species were all clustered as ‘unknown species’, whereas for others only the genus could be determined. Dead biomass was also recorded. Based on this information, we calculated plant species richness, the Shannon diversity (*H’*), and the evenness (*J’*) of the communities [39]. The remaining biomass was dried without separation, providing productivity per EcoUnit.

### (d) Plant performance and functional traits

Plant functional trait data were collected for one species each per functional group of grasses (*Bromus hordeaceus*), non-legume forbs (*Plantago lanceolata*) and legumes (*Trifolium repens*) just before the harvests in October and December. The species were selected based on their frequent occurrence in the EcoUnits. However, not all plant traits were measured on all species and in all EcoUnits. *P. lanceolata* was originally not sown into the communities but had become one of the dominant species in the EcoUnits by October and was thus selected for our experiment. It was not very abundant by the end of the experiment as it did not regenerate well after the harvest in October.

All traits were collected and measured just before the harvest according to [23] unless stated otherwise. Stretched plant height of three representative individuals per species and EcoUnit was measured using a ruler. Then, 10 healthy leaves from at least three manually randomly selected individuals per species and EcoUnit were harvested and transported to the laboratory, where SLA, LDMC, toughness, hairiness and wettability were measured. All 10 leaves were scanned on an Epson Expression 11 000 XL scanner and the resulting images were analysed using imageJ to determine the leaf area. In the case of *T. repens*, only the lamina was scanned. Leaves were weighed and subsequently dried at 70°C for at least 48 h, and dry weight was recorded to calculate SLA (leaf area of fresh leaf/dry weight) and LDMC (dry weight/fresh weight). All weights were measured using a precision scale (QUINTIX315_1S, Sartorius Lab Instruments GmbH & Co. KG, Goettingen, Germany). A few days afterwards, the chlorophyll fluorescence measurements and the SPAD values were determined on living plants in the EcoUnits, just before the harvests.

The hairiness, or rather density of trichomes, of the leaves was analysed by counting the hairs from an image taken at 400-fold magnification using a light microscope and focusing on the middle part of the leaf (Ocular 10x/22, Di-Li-2009, Distelkamp-Electronic, Kaiserslautern, Germany) in ImageJ. For that, four of the leaves used in SLA measurements were chosen at random. Hairs were counted on the upper and lower leaf side and then added to make a total for both leaf sites. *T. repens* did not show any hair on its lamina. The samples of this species were excluded from the subsequent analysis.

The leaf thickness was measured with a digital caliper (WEZU Messwerkzeuge Remscheid GmbH, Remscheid, Germany) at the same spot as leaf toughness. For leaf toughness, the puncture resistance was measured using a surgical blade at a speed of 129 mm min^−1^ on an electric test stand (Sauter GmbH, Wutöschingen, Germany) and the force of the cut was measured with a power meter (FH 50, Sauter GmbH). The leaf toughness was than calculated as the quotient between the puncture resistance and the thickness.

The leaf wettability was investigated via measuring the contact angle (CA) of a water droplet and the leaf, where high CA means low wettability [30]. For that, a droplet of 5 µl distilled water was placed on a flat leaf surface for 90 s and then photographed (Nikon D5300 with a Sigma DC Objective, Chiyoda, Tokio, Japan). The CA was then measured using ImageJ.

Chlorophyll fluorescence was measured using a PocketPEA device (Hansatech, King's Lynn, Norfolk, UK). We measured the parameters PI_abs_ as well as plant stress via F_v_/F_m_ after 30 min of dark adaption to ensure a full reduction of the photosystems on three replicate individuals for each EcoUnit and species [40,41]. These measurements were not performed on *P. lanceolata,* as not many individuals were abundant after harvesting the leaves for the previous analysis.

The SPAD value was measured using a SPAD 502 (Minolta Camera Co., Osaka, Japan) on the same individual. For each individual, three replicate measurements were performed as the values varied within individuals.

### (e) Statistical analysis

To analyse the biomass of the plant communities in relation to changes in ALAN, we used biomass of the EcoUnits as the response variable and ALAN as well as harvest time and their interactions as explanatory variables in a linear model. ALAN was log-transformed prior to all analyses (log_10_ (ALAN + 0.0014)), where 0.0014 lx was the background illumination within the Ecotron facility.

To test whether there were any differences between species, we did the same analysis including species and all twofold interactions as a covariate. We included the species sown (if present) as well as all species found in the EcoUnits in this analysis. We also analysed the proportion of dead plant material.

We used linear models with species richness, Shannon diversity and evenness as dependent variables and ALAN as well as harvest and the interaction thereof as explanatory variables in three separate models, one for each diversity index. To assess whether ALAN influenced the species compositions within the different EcoUnits, we performed a detrended correspondence analysis (DCA) on the species-specific biomasses measured in each EcoUnit and in each harvest. Biomass of the respective species was taken as a proxy for plant abundance, and thus we used it to quantify differences in community composition [42]. We did not include dead plant material in this analysis, as this was not identified by species.

To analyse the effects of ALAN on plant traits, we used linear models with ALAN as explanatory variable and traits (table 1) as dependent variables, respectively. We set up models for each trait separately. We included harvest time as well as species as covariates to evaluate the difference between timing and species-specific differences. All twofold interactions were included in the model. Overall model statistics of the simplified models are reported.

All statistical analyses were done using in R, the DCA was computed using the ‘vegan’ package [43,44] and all models were simplified via bootstrapping [45]. Graphical representations were done using the packages Ggplot2 [46], tidyr [47], dplyr [48] and broom [49].

## 3. Results

### (a) The impact of ALAN on plant community productivity and composition

**Figure 1.**
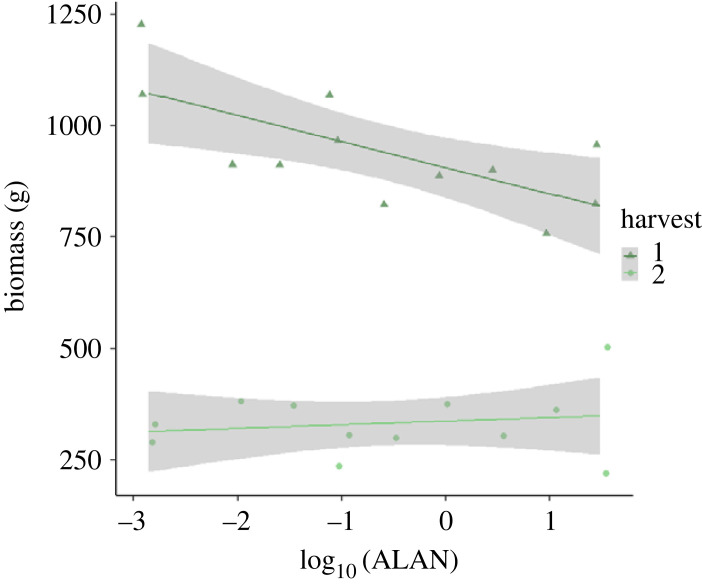
Biomass (g) of the EcoUnits as dependent on ALAN. Dark green triangles and line represent the biomass values after the first harvest in October; light green points describe the biomass harvest in December. Lines are regression lines; the errors of the parameters are depicted as grey bands.

Total plant biomass decreased with increasing ALAN in the first harvest, whereas there was little change in the second harvest (full model: *R*^2^ = 0.94, *F*_3, 20_ = 100.6, *p* < 0.001; figure 1). When looking at the response within species, we saw similar patterns: the biomass decreased with increasing light intensity in the first harvest and had a slightly positive but non-significant slope in the second harvest. All species showed this pattern and the interaction species : ALAN was not significant, thus there was no species-specific response to ALAN in terms of biomass (*R*^2^ = 0.80, *F*_38, 165_ = 17.1, *p* < 0.001; electronic supplementary material, figure S1).

**Figure 2.**
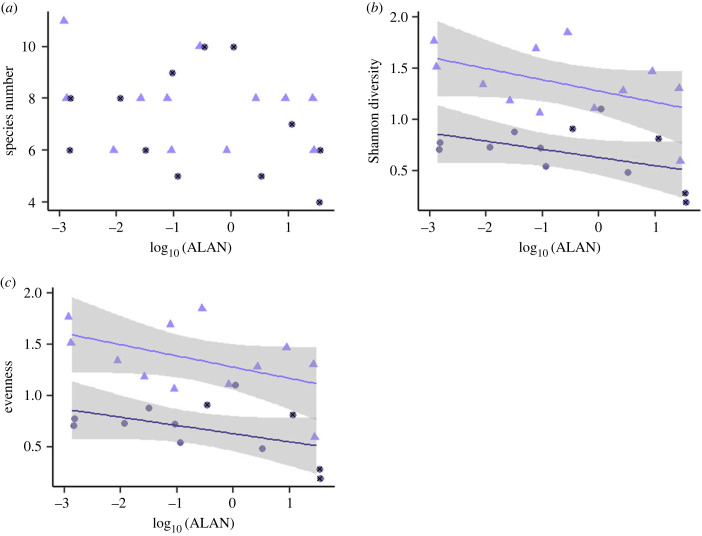
Diversity parameters as dependent on ALAN and harvest time: (*a*) number of species, i.e. species richness, (*b*) Shannon diversity and (*c*) evenness. The EcoUnits are displayed with purple triangles; the darker circles with crosses represent the second harvest. When a relationship was significant, regression lines as based on a linear model are given; the errors of the parameters are depicted as grey bands.

**Figure 3.**
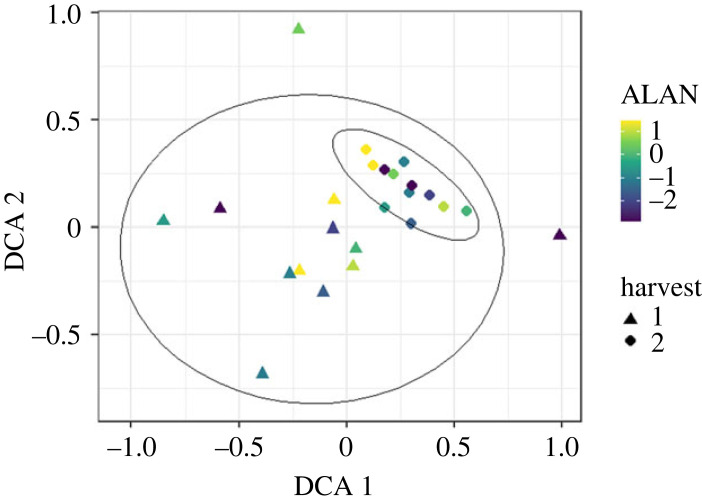
Detrended correspondence analysis (DCA) of the species-specific biomasses in each EcoUnit and between the two harvests. Light intensity of ALAN is colour-coded ranging from low light intensities (purple) to high light intensities (yellow) of ALAN. The two harvests are displayed with different symbols: the October harvest is displayed as triangles; the December harvest is displayed as circles. The Eigenvalue of DCA 1 was 0.27, the Eigenvalue of DCA 1 was 0.14. Ellipses show 95% confidence interval for each sample date.

A few species, namely *Campanula patula* L., *Cynosurus cristatus* L*.* and *Plantago media* L. did not survive and/or germinate in our experiment (see electronic supplementary material, table S1). Alongside our sown species, *Festuca pratensis* Huds., *Festuca* sp., *Holcus lanatus* L., *Medicago sativa* L., *P. lanceolata*, *Poa* sp., *Poa pratensis* L., *Poa trivialis* L., *Silene vulgaris* (Moench) Garcke, *Stellaria* sp., *Trifolium pratense* L. and some undetermined species occurred in the EcoUnits. Some species like *Prunella vulgaris* L., *Ranunculus repens* L. and *Veronica chamaedrys* L. were noticeably absent under higher ALAN, and *Medicago lupulina* L., *V. chamaedrys* and *Vicia sepium* L. could not be found in the second harvest at all (electronic supplementary material, figure S1 and table S1). We did not find any significant effect of ALAN on plant species richness as the artificial meadows were species-poor, but Shannon diversity and evenness declined significantly with increasing ALAN. This decline was consistent across both harvests, whereas Shannon diversity and evenness were a bit lower in the second harvest compared to the first harvest (*R*^2^ = 0.66, *F*_2, 21_ = 20.5, *p* < 0.001 and *R*^2^ = 0.73, *F*_2, 21_ = 29.7, *p* < 0.001, respectively; figure 2). Plant communities in the second harvest were more similar to each other, yet they were not different from the communities in the first harvest, as the ellipses designating 95% confidence interval for each sample date overlapped (figure 3).

### (b) Plant traits

**Figure 4.**
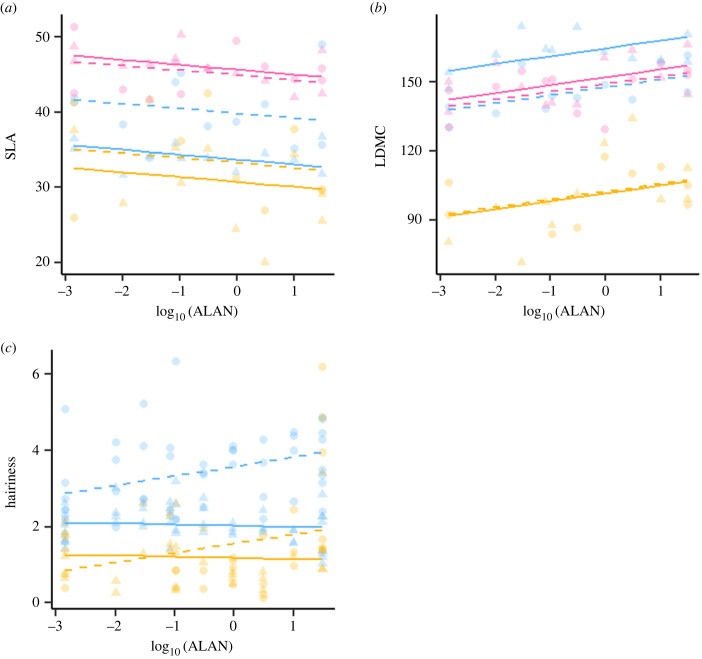
Effects of ALAN on plant functional traits. Displayed are also the differences between the selected species and the two harvests. (*a*) Specific leaf area (SLA), (*b*) leaf dry matter content (LDMC) and (*c*) hairiness. The darker shade of the colours denotes the earlier harvest (October), the lighter the later one (December). *Bromus hordaceus* is depicted in blue, *Trifolium pratense* in pink and *Plantago lanceolata* in gold. Regression lines were drawn in the graphs in the respective colours.

ALAN had significant effects on some plant traits (3 out of 11 traits considered), but these effects varied across species and harvests (figure 4). Plant height was not affected by ALAN, but more intense night light exposure reduced SLA. This was true for all species, yet the SLA differed between harvests and species (*R*^2^ = 0.69, *F*_6, 61_ = 23.1, *p* < 0.001; figure 4*a*). The species effect also differed between the harvest times, but all species in both stages showed increasing LDMC with increasing ALAN (*R*^2^ = 0.86, *F*_6,61_ = 61.9, *p* < 0.001; figure 4*b*). The hairiness showed increases and decreases with increasing ALAN, and the magnitude of the slope depended on species identity and harvest time (*R*^2^ = 0.52, *F*_5,148_ = 31.5, *p* < 0.001; figure 4*c*). ALAN had no significant influence on wettability, thickness, toughness, F_v_/F_m_, PI_abs_ or SPAD.

## 4. Discussion

In this highly controlled Ecotron study, we showed that plant community biomass as well as plant species-specific biomass were strongly affected by ALAN, with a 33% decrease in plant biomass at the October harvest in the largest ALAN treatment, when biomass was comparably high. We did not detect an ALAN effect on community composition using a DCA. Nonetheless, ALAN decreased the Shannon diversity by 43% and evenness by 34% in the first harvest, while plant species richness was consistent with the null hypothesis. Importantly, not all species grew in every experimental community; some species were absent from high ALAN conditions, whereas others occurred in all treatments. Moreover, ALAN shifted the expression of some plant traits related to plant performance. Considered together, decreases in biomass, diversity and evenness, and shifts in plant traits suggest that plant communities display an increasing stress response along our experimental gradient in ALAN.

Plant biomass decreased with increasing ALAN in all the species studied in the first harvest. This is quite different from [17], which found strong species-specific differences in natural grassland communities near imposed experimental light treatments, 1 m above the ground. The light intensity within the EcoUnits, however, never reached the outside light conditions of a full, sunny day (light saturation of most plant species is between 500 and 1200 µmol m^−2^ s^−1^); it may therefore be the case that our plants were low-light adapted in general, and thus responded in a more pronounced way to ALAN-induced stress. We chose this approach though to create a gradient in ALAN, while controlling for other parameters, avoiding (for instance) seasonal drought effects or biomass removal due to herbivores or disturbance. Even though light levels at night were high (around 2% of the daytime illumination within the EcoUnits under the highest ALAN treatment and full moon), they suffered rather than benefited from it. The consistent decrease in productivity across species in plant communities subjected to high levels of ALAN is remarkable, especially given that plants were not likely to be able to use the light at night to activate photosynthesis [18].

An important caveat is that we detected a strong influence of harvest time on many response variables, including plant biomass, and some plant traits. Notably, the experimental duration between harvests was longer before the first compared to the second harvest. Therefore, the plants may not have been as well developed and were in general shorter in harvest 2. The effects of ALAN were much stronger on the plants that had a longer time to grow before the first harvest than on plants assessed in the second harvest, suggesting that cumulative effects of ALAN may be realized earlier in the season or after longer growth periods. Daylength was shorter in December than in October because we mimicked outside conditions, so the time for photosynthesis was also reduced. Additionally, the temperature was slightly colder, yet above outside conditions (mean soil temperature (20 cm below surface) between all EcoUnits was 17.3°C in October and 16.7°C in December). Also, the noticeable absence of some plant species in the second harvest could be due to the fact that they did not cope with the disturbance induced by the harvest itself.

The community composition was more affected by season of harvest than by the different ALAN regimes. In general, plant communities in the first harvest were much more diverse between as well as within communities than in the second harvest, which can be seen in the spread within the DCA as well as the Shannon diversity. The decline in evenness as observed in this study further illustrates the effects of ALAN, as some species became dominant and others failed to reestablish after the harvest. It is striking to see that some species failed to grow in the high-ALAN treatments (see electronic supplementary material, figure S1), but also fewer species from the local seedbanks were successful in germinating and growing in high ALAN treatments. This cannot be explained by heterogeneous starting communities, as the soil was thoroughly mixed through before putting it into the EcoUnits. Whether species absence was due to unfavourable germination conditions or poor growth and competitive ability in the communities subjected to high ALAN cannot be deduced from the data we collected, as we only have information on plant biomass at the time of harvest. However, other studies also found this effect, which was noticeably reduced in invasive grass species [50,51].

Plant height did not respond significantly to ALAN, whereas plant biomass consistently decreased with increases in ALAN in all the species studied, irrespective of their dominance or height. This implies that, although not assessed, plant width and/or frequency must have decreased due to inhibited growth or germination. Possibly, the plants altered their above-ground–below-ground allocation of vegetative tissue in response to changes in the light environment. Typically, such growth allocation patterns are reported in response to increased light during the day, which is considered as beneficial rather than detrimental to plants [52]. Importantly, the growth-related traits SLA and LDMC, as well as the hairiness, changed in a significant way within species with changes in ALAN. SLA decreased, whereas LDMC and hairiness increased. This shows an intraspecific change associated with a reduction in plant growth that could be related to an increase in resistance (indicated by higher LDMC) and light protection, which is provided by hairs [23,25,26]. This can again be a seasonal effect, as species increase LDMC and decrease SLA in autumn if leaves are not shed [53,54]. The increase in hairiness was especially noticeable in harvest 2, which could be due to the fact that they help the plant to insulate against cold temperatures and not just against high irradiation [26,52]. It is notable though that the leaf thickness did not increase, which could be the driving factor for the lower SLA and higher LDMC. Thus, the light presumably did not trigger the growth of multiple layers of palisade parenchyma, as could have been expected from plants growing at higher light conditions [52]. This could perhaps be explained by an increase in cell wall thickness and other compounds leading to higher weight per area and, thus, higher resistance [23]. Chlorophyll fluorescence parameters and chlorophyll content did not change throughout the experiment in response to ALAN, i.e. the fitness of the individual plants was consistent, as was the performance [41,55,56]. Previous studies showed a strong decrease in F_v_/F_m_ and PI_abs_, with senescence effects at the end of the season [53,54,57]. Thus, senescence effects within the remaining plant species can be excluded as driving factor between patterns. Possibly, these effects were even counteracted by ALAN, which is known to extend growing season length [58]. More research is needed to evaluate mechanisms underlying these biomass responses to ALAN. It would be particularly valuable to examine patterns and timing of plant resource allocation (above-ground versus below-ground biomass, or vegetative versus reproductive tissue).

## 5. Conclusion

We found that ALAN negatively affected plant biomass, diversity, and some plant functional traits under the controlled conditions of the iDiv Ecotron. These are the first results on how plant communities respond to a gradient of ALAN, providing further insights than a simple comparison of growth with or without ALAN. From previous research, we know that substantial changes in plant communities such as observed here will likely have significant cascading effects on other organisms above and below the ground (e.g. [37,59–61]), as well as on multiple ecosystem processes [61–63] and plant community resistance (e.g. [64]). These results suggest a need to examine the impact of light on plant communities in more detail because most of our land surface is susceptible to ALAN, and the impact and extent of ALAN are still increasing on continental and global scales [1,3]. This has major implications for plant conservation and the establishment of protected areas far from human impact and it illustrates that measures to reduce ALAN should be considered, such as avoiding the direct illumination of trees and roadside areas where plants are present.

## Data Availability

The data are provided in electronic supplementary material [65]. Data are also included from the Dryad digital repository: https://doi.org/10.5061/dryad.hhmgqnknt [66].
